# Short introduction to JAHEE and Actions

**DOI:** 10.1093/eurpub/ckac129.433

**Published:** 2022-10-25

**Authors:** G Costa

**Affiliations:** Piemonte Region, Turin, Italy

## Abstract

Reducing health inequalities is on the agenda of many countries. Despite an increasing concern and awareness on health inequalities a wide gap exists in Europe in terms of political response. The main objective of JAHEE was to strengthen a cooperative approach among participating countries and implement concrete actions to reduce health inequalities. The partnership was composed of 24 countries including many strategically most relevant public health institutions in the European Union, which contributed with different backgrounds, skills and know-how to the achievement of the project objectives. The main results will be presented.

